# Supplementary material for: The Systems Biology Research Tool: evolvable open-source software
Source: BMC Syst Biol. 2008 Jun 29;2:55. doi: 10.1186/1752-0509-2-55 (PMC2446383; doi:10.1186/1752-0509-2-55)
Supplement: Additional file 1 — SBRT Archive. An archive of the current version of the Systems Biology Research Tool. [file 1752-0509-2-55-S1.zip › sbrt-1.4.0/doc/developers_guide/api/sbrt/kernel/math/geom/comp/package-use.html]

Uses of Package sbrt.kernel.math.geom.comp


|  |  |  |
| --- | --- | --- |
| **Overview** \| **Package** \| Class \| **Use** \| **Tree** \| **Deprecated** \| **Index** \| **Help** | |  |
| PREV   NEXT | **FRAMES**    **NO FRAMES**     **All Classes** |  |


---


## **Uses of Package sbrt.kernel.math.geom.comp**

| Packages that use sbrt.kernel.math.geom.comp | |
| --- | --- |
| **sbrt.kernel.math.geom.comp** | Provides classes and interfaces for performing geometry-related computations. |
| **sbrt.kernel.math.geom.proc** | Provides classes and interfaces for performing geometry-related processes. |

Classes in sbrt.kernel.math.geom.comp used by sbrt.kernel.math.geom.comp

****CdHarComputer.Results****
  
          This class is used to represent the results of a random point
computation.

Classes in sbrt.kernel.math.geom.comp used by sbrt.kernel.math.geom.proc

****CdHarComputer.Results****
  
          This class is used to represent the results of a random point
computation.

---


|  |  |  |
| --- | --- | --- |
| **Overview** \| **Package** \| Class \| **Use** \| **Tree** \| **Deprecated** \| **Index** \| **Help** | |  |
| PREV   NEXT | **FRAMES**    **NO FRAMES**     **All Classes** |  |


---
